# Upregulation of Stromal Cell–Derived Factor 1 (SDF-1) is Associated with Macrophage Infiltration in Renal Ischemia-Reperfusion Injury

**DOI:** 10.1371/journal.pone.0114564

**Published:** 2014-12-05

**Authors:** Xin Wan, Wenkai Xia, Yasser Gendoo, Wen Chen, Wenjin Sun, Dong Sun, Changchun Cao

**Affiliations:** 1 Department of Nephrology, Nanjing First Hospital, Nanjing Medical University, Nanjing, Jiangsu, China; 2 Department of Thoracic and Cardiovascular Surgery, Department of surgery, Nanjing First Hospital, Nanjing Medical University, Nanjing, Jiangsu, China; Indiana University School of Medicine, United States of America

## Abstract

**Background:**

Stromal cell-derived factor-1(SDF-1) is a chemotactic and angiogenic factor that mediates the repair of various tissues. As macrophages are important contributors to ischemic kidney injury, we examine the role of SDF-1 in a rodent model of ischemia-reperfusion (I/R) injury.

**Methods:**

Male wild-type (WT) (C57BL/6) mice were subjected to bilateral I/R injury or sham operation in the presence or absence of macrophage depletion (liposomal clodronate [0.2 ml/20–25 g body weight i.p.]). Macrophage accumulation was assessed by immunohistochemistry. Tissue levels of SDF-1 (ELISA) and SDF-1 mRNA expression (real-time PCR) were measured. The cellular location of SDF-1 was assessed using immunohistochemical staining.

**Results:**

Immunofluorescence staining of renal tissue sections confirmed macrophage depletion by liposomal clodronate. SDF-1 production was elevated in response to I/R injury and was significantly increased upon macrophage depletion. SDF-1 positive cells initially appeared initially in the cortex, and subsequently diffused to the outer medulla after I/R injury.

**Conclusions:**

Our study demonstrates that SDF-1 is significantly upregulated during renal I/R. We hypothesize that SDF-1 upregulation may be an important macrophage effector mechanism during I/R injury.

## Introduction

Acute kidney injury (AKI) induced by ischemia is accompanied by a relatively inefficient recovery capacity of the kidney [1]. Given that renal injury interferes with normal tubular epithelial cell (TEC) integrity, this ultimately leads to impaired maintenance of water and electrolyte homeostasis as well as reduced excretion of metabolic waste. Thus, mechanisms and mediators of tissue repair have evolved to rapidly cover the area of injury and initate cellular differentiation and tubular regeneration. Kidney repair is comprised of surviving epithelial cells that adapt to the loss of adjacent cells through proliferation to restore cell number and permit inflammatory cell infiltration [2]. These phases of tissue repair result in the restoration of the functional integrity of the nephron [3]. In addition, the inflammatory phase of repair, initiated a few hours after injury by the infiltration of immune cells from the kidney, is of central importance to kidney regeneration.

In the early phase of renal I/R injury, monocytic phagocytes are detrimental because they promote apoptotic cell death by releasing reactive inflammatory mediators [2]. Tissue injury is a complicated and highly regulated process coordinated by a large number of growth factors, chemokines and cytokines, all of which are derived from normal or damaged tissue [4]. Although a selective approach to macrophage depletion can occassionally reduce AKI by attenuating inflammation and the ensuring fibrosis, the mechanisms by which macrophages contribute to tissue injury are largely unknown and demand attention.

Recently, several studies have suggested a significant role for chemokine stromal cell-derived factor-1 (SDF-1 also known as CXCL12) in mediating tissue repair by promoting the migration of circulating stem or progenitor cells to sites of ischemic injury to facilitate repair [5], [6]. Contrary to the selective induction of most chemokines by certain stimuli, SDF-1 is up-regulated in a variety of damaged tissues and organs as part of the injury or DNA damage response. In addition, SDF-1 is highly inducible in several pathologic conditions including ischemia and/or hypoxia and in angiogenic environments [7], [8], [9], [10], [11]. Binding of SDF-1 to CXC chemokine receptor (CXCR) 4 activates multiple downstream pathways that mediate stem/progenitor cell chemotaxis and organ-specific homing in ischemia-reperfusion tissue [12], [13], [14], and this occurs through the activation of NF-κB, the Erk/ELK pathway and JAK/STAT signaling [15], [16], [17]. The great importance of SDF-1 in cardiac, neuronal and hematopoiesis development is further emphasized by the fetal death of either SDF-1 or CXCR 4 knockout mice [18]. In a model of I/R injury induced renal neovascularization, SDF-1 levels were elevated in the kidney, and administration of an anti-SDF-1 antibody severely increased renal dysfunction and injury [19].

To further improve our understanding of renal ischemia inflammatory processes, we used a renal ischemia-reperfusion model to investigate the *in vivo* expression of SDF-1. Meanwhile, depletion of macrophages before I/R resulted in less severe injury accompanied by significantly increased SDF-1 expression, improved tubular cell proliferation and decreasing morphologic kidney damage during the inflammatory phase of increased macrophage presence.

## Materials and Methods

### Ethics statement

All mice were housed in specific pathogen-free cages at 23 ± 2°C and 60 ± 10% humidity, with a 12-hour light/12-hour dark cycle and free access to food and water. All animal procedures were performed in compliance with the Institute of Laboratory Animal Research Guide for the Care and Use of Laboratory Animals of the National Institutes of Health and were approved by the Institutional Animal Care and Use Committee of Nanjing Medical University.

### 1. Mice and ischemia/reperfusion (I/R) model

Six- to eight-week-old wild-type male C57BL6 mice were purchased from the Model Animal Research Center of Nanjing University (Nanjing, China). Induction of kidney IRI was carried out as described previously [20]. Briefly, Mice were anesthetized with chloral hydrate and left renal pedicles were clamped for 30minutes. After removal of the clamps, blood flow was re-established with the kidneys returning to original color. Sham-operated animals received similar surgical procedures with the exception of the renal pedicles and were sacrificed at day 1 after surgery. During the surgical procedure, body temperature was maintained around 37°C. Animals were sacrificed with cervical dislocation at day 1, 3 or 7 following reperfusion, blood was collected, and kidney samples were harvested and stored at -80°C for further analysis.

### 2. Liposome preparation and administration

Liposomal clodronate (LC) and liposomal vehicle (LV) were prepared according to previously described methods [21]. Mice received an intraperitoneal bolus 0.12-0.2mL/20-25g body weight of vehicle or clodronate encapsulated liposomes (Formumax, USA) 48 prior to I/R injury, as previously reported by others [22].

### 3. Quantification of mRNA by real-time PCR

Total RNA was isolated from kidneys and reverse transcribed using the PrimeScript^TM^ RT reagent Kit with gDNA Eraser (TaKaRa, Dalian, China). cDNA was amplified using SYBR Premix Ex Taq^TM^ II (TaKaRa, Dalian, China) and the Applied Biosystems 7500 sequence detection system (Applied Biosystems, USA). The primers used were: SDF-1 (forward: 5′-TGAGGCCAGGGAAGAGTGAG -3′; reverse: 5′-GACACATGGCGATGAATGGA-3′). Relative expression was calculated using the comparative threshold cycle (Ct) method. All samples were normalized to GAPDH.

### 4. Assessment of renal function

Formalin-fixed kidney sections of 5-µm thickness were cut and stained with haematoxylin and eosin (H&E). Injury to tubules was graded using a semi-quantitative scale from 0 to 5,where 0 = none, 1 = <10%, 2 = 11% to 25%, 3 = 26 to 50%, 4 = 51 to 75% and 5>75%. Determination of serum creatinine was performed using the analyzer in the diagnostic laboratory.

### 5. Immunohistochemistry

Renal tissue sections were subjected to immunohistochemical staining for SDF-1 and CD68 (ED1). For immunohistochemical staining, 3 µm renal sections were deparaffinized and rehydrated in graded alcohol. The sections were immersed in 3% hydrogen peroxide for 10 min to block endogenous peroxidase activity and were then incubated in buffered normal horse serum to block non-specific binding. Prior to immunochemistry, sections were subjected to antigen retrieval by immersion in a 0.1 mol/L citrate buffer (pH 6.0) for 25 minutes, followed by heating in an electrical pressure cooker for 5 minutes. Sections were incubated overnight at 4°C with rabbit polyclonal anti-SDF-1 antibody (1:100, eBioscience, San Diego, CA, USA) and mouse monoclonal anti-CD68 antibody (1:100, Abcam, USA), both of which were primary antibodies. Control experiments included omission of either the primary or secondary antibody. The next day, sections were incubated with horseradish peroxidase (HRP)-conjugated goat anti-rabbit or rat anti-mouse secondary antibody (Beijing Zhongshan Biotechnology Co., Beijing, China) for 1 h at room temperature. Then, 3, 3-diaminobenzidine tetrahydrochloride (DAB, Beijing Zhongshan Biotechnology Co., Beijing, China) was applied to the slides to develop a brown color. Counterstaining was performed with hematoxylin, and photomicrographs were taken with an Olympus camera (Olympus BX51, Japan).

### 6. SDF-1 quantitation

The expression of SDF-1 in kidneys from each group was measured by the SDF-1 Rapid ELISA Kit (HUIJIA Biotechnology, Xiamen, China) according to the manufacturer's protocol.

### 7. Statistical analysis

The results are presented as means ± SD. The Wilcoxon test was used for non-parametric data analysis. When normal distribution of data were assessed with the Kolmogorov-Smirnov, statistical comparisons between experimental groups were evaluated by the student's t test and one-way ANOVA using SPSS 20.0 software (SPSS Inc, Chicago, IL, USA); a *P* value <0.05 was considered significant.

## Results

### 1. Kidney function and histological changes after IR injury

**Figure 1:**
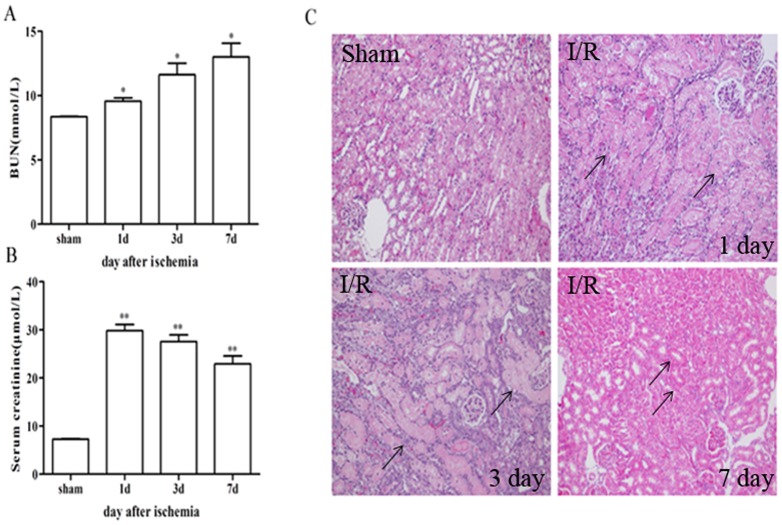
Kidney tissue injury over time following 30min of bilateral renal ischenmia-reperfusion injury. C57BL/6 mice were subjected to sham or bilateral ischemia by clamping the renal pedicles for 30 min and then removing the clamps and confirming reperfusion. Mice were sacrificed at various times and kidney samples were collected. (A and B) BUN and serum creatinine were measured to determine renal function.The data shown were the means±SD. n = 6 per group. *P <0.05, vs sham; **P <0.01, vs sham(C) Photomicrograps of H & E-stained kidney sections (200×). All fields were chosen form cortex and outer medulla. Tubular damage is marked with arrows.

Mice subjected to I/R injury-induced tubular cell death exhibited a loss of glomerular filtration accompanied by a rapid increase in serum creatinine concentration (Scr) and BUN level (Figure 1A and 1B). Histological analysis of H&E-stained kidney sections of the corticomedullary region was performed on day 1, 3, and 7 post-reperfusion (Figure 1C). Widespread damage was noted in bilateral ischemic forms of AKI following IR injury in the form of dilated, flattened, and swollen epithelium and loss of proximal tubular epithelial cells with luminal cast formation.

### 2. Localization of SDF-1 protein in the kidney

**Figure 2:**
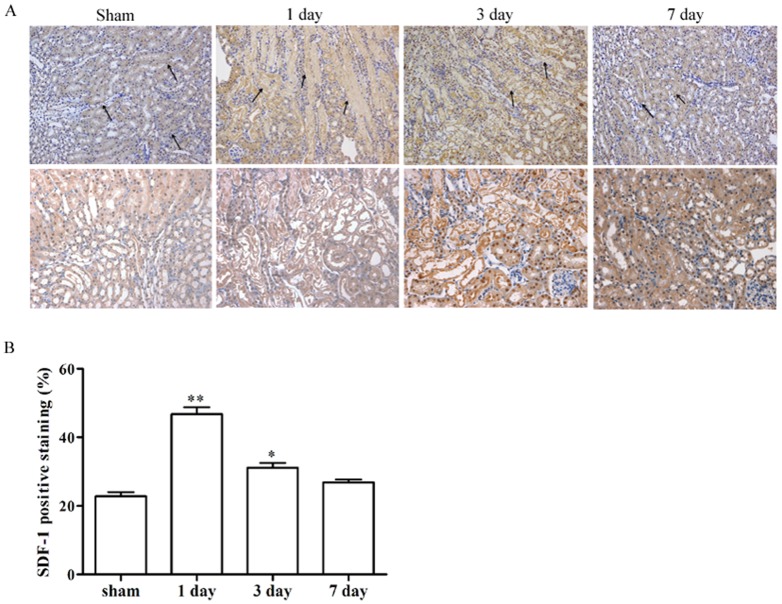
Regional location of SDF-1 in I/R kidney. (A) Immunohistochemistry staining of SDF-1 in the kidney also showed that IR-induced expression of SDF-1 was further distributed into the surrounding corticomedullary and outer medullary region compared to sham-operated mice. The kidney sections from sham-operated mice were used as control. (upper panels original magnification 200×; bottom panels 400×). (B) Quantification of SDF-1 positive area. Values are means ± SD. **P <0.01, vs sham.

In order to determine expression of SDF-1 in kidneys obtained from mice submitted to sham or I/R surgery, we subjected tissue sections to immunohistochemical analysis (Figure 2A and 2B). The results showed that SDF-1 in the sham-operated kidney was readily detected in both the cortex and medulla. It was markedly greater in the cortex, except for in glomeruli, where it was completely absent. However, its presence sharply increased and invaded the surrounding corticomedullary and outer medullary region during ischemia-reperfusion injury.

### 3. Expression of SDF-1 in the kidney following I/R injury

**Figure 3:**
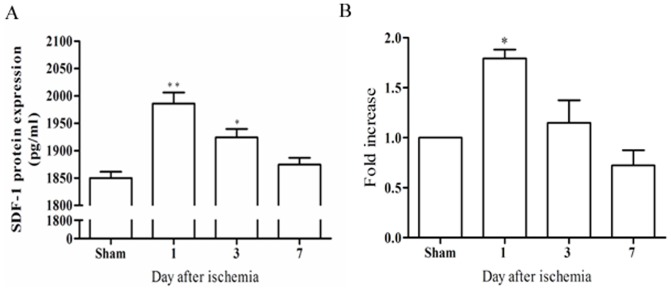
SDF-1 protein levels after ischemia-reperfusion (I/R) injury. (A)Levels of SDF-1 were determined by ELISA using whole-kidney homogenates obtained from sham-operated animals or animals sacrificed at Day 1, 3 or 7 following ischaemia. The amount of SDF-1 at Day 1 was significantly higher compared with that found in sham-operated mice, and with still significantly elevated levels at day 3. Values are means ± SD; n = 6 per group. **P <0.01, vs sham; *P <0.05, vs sham. (B) SDF-1 mRNA levels after ischemia-reperfusion (I/R) injury. Real-time polymerase chain reaction (PCR) quantification of SDF-1 mRNA showed a significant increase at day 1 after ischemia. Values are means ± SD; n = 6 per group. *P <0.05, vs sham.

To detect the renal SDF-1 production during different phases of renal ischemic injury, mice were subjected to bilateral I/R injury and were sacrificed at various times. Active renal SDF-1 production remained low in sham-operated animals. However, it significantly increased on day 1 in response to I/R injury and decreased steadily in the following days (Figure 3A). To provide more detailed evidence, renal SDF-1 mRNA expression was quantitatively assessed using real-time PCR. High levels of SDF-1 mRNA expression were detected 24 h post-injury followed by a decrease over the following days (Figure 3B).

### 4. Macrophage infiltration during I/R injury

**Figure 4:**
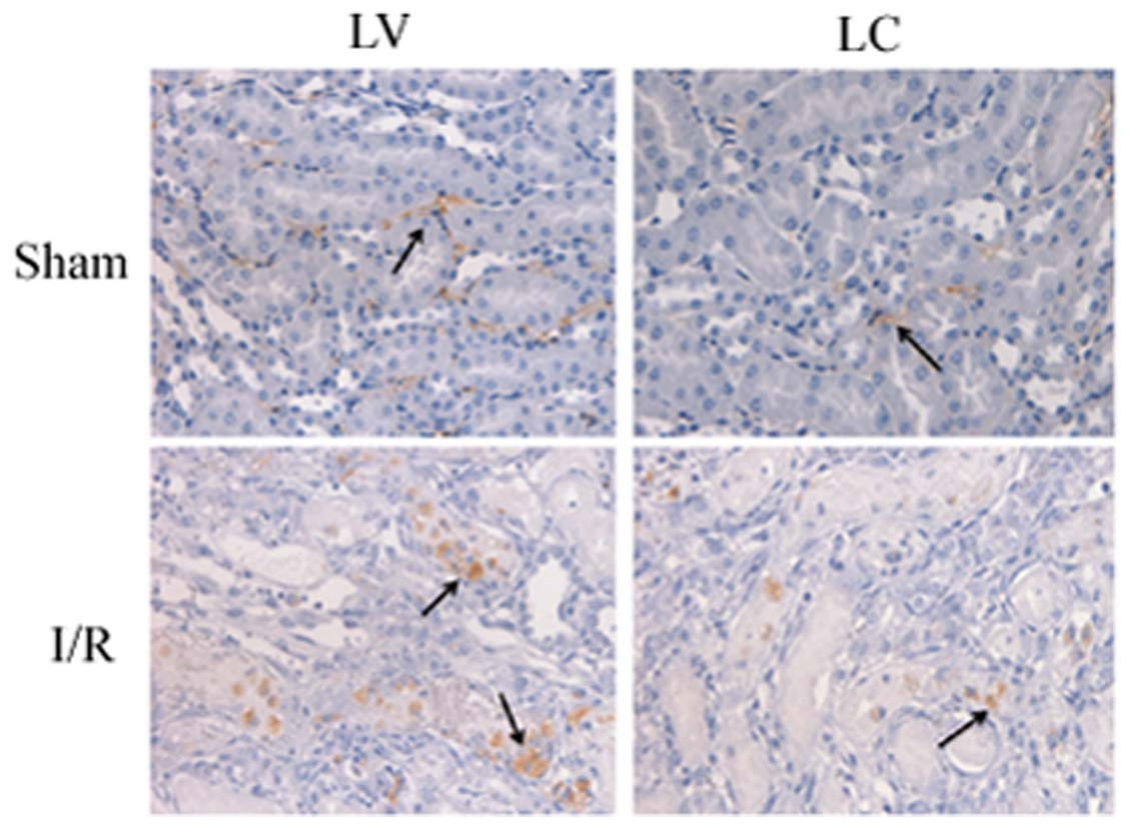
Proinflammatory macrophages accumulation following I/R. Photographs depicting macrophage in LV and LC treated mice exposed to sham operation or I/R. (arrows, 400×).

Renal cortical tissue sections were stained with an antibody specific for mouse macrophage marker CD68 to assess the degree of macrophage infiltration during renal ischemia-reperfusion injury. Sham-operated mice exhibited minimal interstitial macrophage staining. Mice that were subjected to reperfusion, demonstrated significant macrophage accumulation after 24 h. The degree of ischemia-induced macrophage infiltration was significantly reduced in the presence of LC, thereby demonstrating effective macrophage depletion in this model (Figure 4).

### 5. LC treatment reduced renal damage and dysfunction during I/R injury

**Figure 5:**
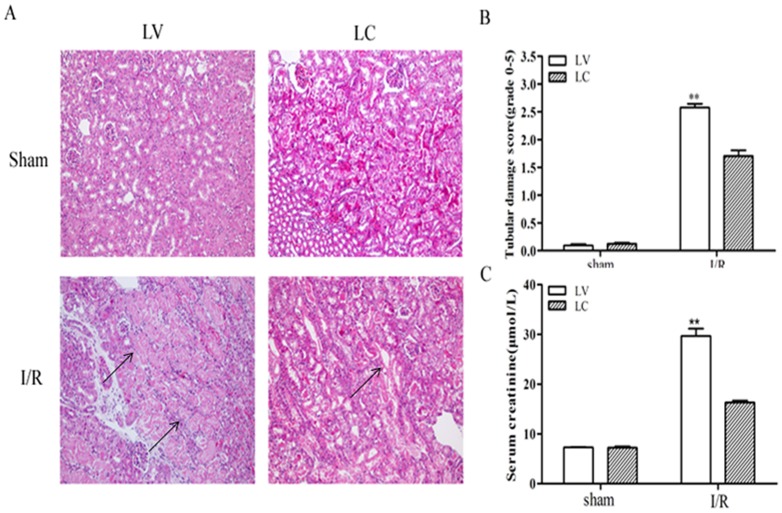
Tubular injury is attenuated in LC-treated mice (A) Histology of mice shows increased tubular injury in the LV+I/R group compared with LC+I/R. (200×). Tubular damage is marked with arrows.(B) Semiquantitative analysis of tubular damage in LC-treated and LV-treated kidney at 24h after reperfusion. Values are means ± SD; n = 6 per group. *P <0.05, vs I/R+LV. (C) Serum creatinine values are shown 24hours after I/R±macrophage infusion. **P <0.01, vs I/R+LV.

Histological examination (Figure 5A) and tubular damage score (Figure 5B) of samples 24 h after I/R injury confirmed that tubular damage was more severe in the LV+I/R-treated animals compared with LC+I/R. LV+I/R treatment resulted in an increase in Scr 24 h post-injury that was significantly worse than LC+I/R (Figure 5C). These data suggested that depletion of pro-inflammatory macrophages reduces renal tubular damage and renal dysfunction after I/R.

### 6. Upregulation of SDF-1 expression after macrophage depletion

**Figure 6:**
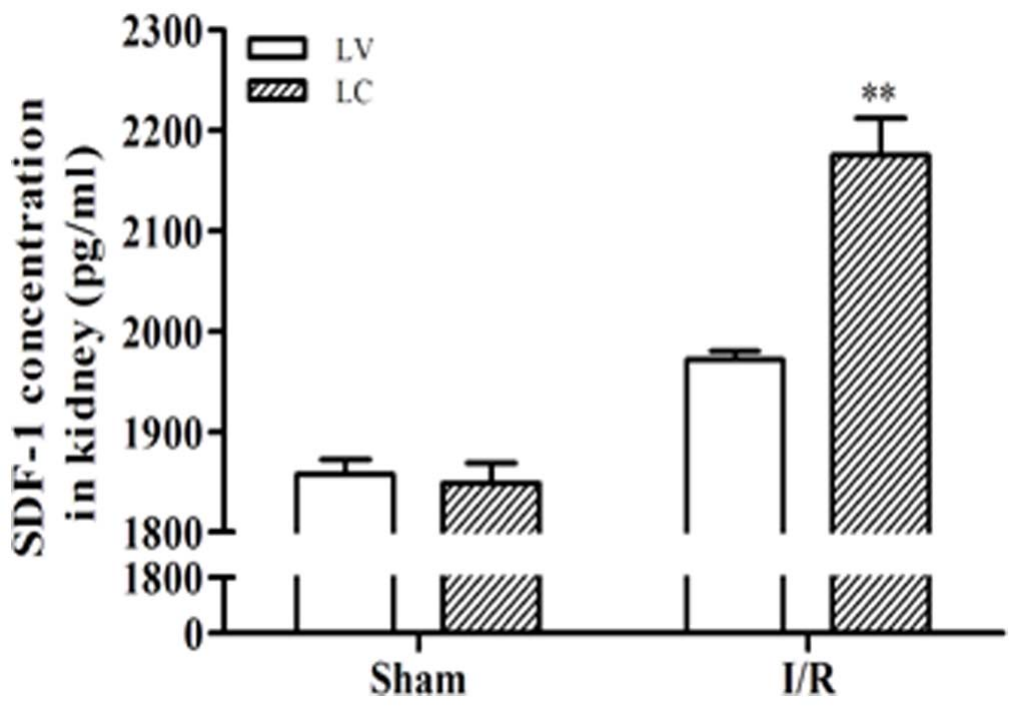
Renal SDF-1 protein levels following LC treatment. Kidney homogenates from mice subjected to I/R injury and treated with LV or LC were analysed for SDF-1 protein using ELISA. LC treatment resulted in an increase of SDF-1 levels compared with the concentration found in homogenates from LV-treated animals that reached statistical significance (n  =  4-6 per group, **P <0.01). Animals were sacrificed 24 h following ischaemia.

Because the highest total SDF-1 levels were found on day 1, we subsequently studied the expression of SDF-1 at this time point. Forty-eight h prior to the onset of I/R injury, mice were treated with either LC or LV (vehicle-treated animals) (Figure 6). ELISA analysis of whole kidney homogenates demonstrated that total SDF-1 concentrations were markedly different between macrophage-depleted and vehicle-treated animals.

## Discussion

Renal ischemia-reperfusion is a major cause of cellular pathology and accounts for the majority of AKI. It is a complex physiological and histological process, resulting in tubular dilation and necrosis, apoptotic cell death, tubular cell proliferation and ultimately renal dysfunction [3]. The regions of the kidney most prone to ischemic injury are the corticomedullary and outer medullary areas, as they are especially sensitive to decreases in oxygen concentration [3], [23]. Ceradini et al. revealed that SDF-1 expression was directly proportional to reduced local tissue oxygen tensions [12].

The initial inflammatory phase drives tissue damage by providing directional signals for the migration and aggregation of macrophages into the corticomedullary junction. However, when the inflammatory phase is prolonged, it can severely disturb kidney recovery processes, and must therefore be tightly controlled [2]. Chemokines are important inflammatory mediators and are involved in a large number of critical functions. Recent studies have shown that SDF-1 participates in the progression and regeneration of inflammation associated with several pathologic conditions [6], [24], [25] by mediating circulatory stem and progenitor cell migration following I/R injury independent of hematopoietic stem cell (HSC) migration [19].

Studies exploring SDF-1expression in the kidney have thus far only been performed on the developing kidney; therefore, the recovery potential of SDF-1 in kidney is further warranted [26]. We and others found that the total amount of SDF-1 in the kidney increases profoundly after renal I/R injury combined with the administration of specific antisense oligonucleotides (ASON). Stokeman et al. demonstrated that SDF-1 inhibition contributes to renal hypoxic injury and dysfunction [19]. This is in agreement with findings that SDF-1 can suppress apoptosis or necrosis and has a protective role during I/R injury [27], [28]. Furthermore, when studying additional time points, we found that SDF-1 was particularly increased during the early phase of I/R injury du ring which the predominant inflammatory response occurs. This suggests that the initial inflammatory reaction for renal injury is likely to determine SDF-1 activation, which plays a larger role in acute ischemia than chronic inflammatory conditions.

In our study, we observed that SDF-1 diffused from the renal cortex to the corticomedullary area following I/R, which confirms the findings that TEC are involved in the generation of SDF-1 in ischemia-induced kidney injury [29]. Meanwhile, up-regulation of SDF-1 gene and protein expression was detected in the early phase of renal ischemia injury. The physiological mechanisms underlying the localized expression of SDF-1 in injured kidneys are not completely understood.

The main focus of our study was to investigate the factors possibly involved in regulating SDF-1 expression during ischemic reperfusion injury. There is compelling evidence suggesting that mononuclear phagocytes/macrophage are one of major cell types that accumulate around interstitial tissue [30]. The time course of up-regulation of inflammatory macrophages (which is responsible for the augmented inflammatory response) and their location of infiltration were similar to those observed for SDF-1.

Our findings also led us to identify a possibly new observation between macrophages and SDF-1. Administration of liposomal clodronate before ischemia-reperfusion significantly attenuated proinflammatory macrophage influx into the kidney and diminished tubular injury. Interestingly, there was also a significant increase in SDF-1 at the same time, indicating that proinflammatory macrophage may inhibit its expression. It is possible that increased apoptotic TEC, in kidneys not treated with liposomal clodronate, increased the signals for macrophages to infiltrate into the kidney to combat infection rather than to modulate the inflammatory response and promote tissue repair.

Among the chemokines expressed in the tissue ischemic microenvironment, SDF-1 has emerged as an important attractant of reparative cells by participating in the recruitment of monocytes and driving macrophage differentiation. This is suggested by the SDF-1-induced up-regulation of crucial angiogenic factors, such as chemokine (C-C motif) ligand (CCL) and VEGF [31]. There is accumulating evidence of an autocrine positive feedback loop in the recruitment and retention of CXCR4+ bone marrow-derived cells that support revascularization of tumors [6], [32], a process that shares features with tissue regeneration. It has been recently reported that a CXCR4 antagonist prevented the influx of monocytes and inhibited tumor growth, resulting in abrogation of glioblastoma recurrence after irradiation in mice [32].

In conclusion, the data show for the first time, that SDF-1 expression in vehicle treated kidneys differs significantly from that of macrophage-depleted kidneys. The data also identify a previously unknown location for SDF-1, namely, the renal corticomedullary region. However, the mechanism by which SDF-1 activates the signaling pathway needs further investigation. We hope that our study will contribute to a better understanding of the complex events involved in renal ischemia-reperfusion, which is key for the development of more effective therapeutic strategies.
